# Isolated splenic metastasis from primary fallopian tube carcinoma and the application of laparoscopic splenectomy: a case report and literature review

**DOI:** 10.3389/fonc.2023.1079044

**Published:** 2023-05-03

**Authors:** Dongxue Kang, Danyang Zhao, Xiaodi Jiang, Deming Li

**Affiliations:** ^1^ Department of Operating Room, The Fourth Affiliated Hospital of China Medical University, Shenyang, China; ^2^ Department of Emergency, The Fourth Affiliated Hospital of China Medical University, Shenyang, China; ^3^ Department of Infectious Diseases, Shengjing Hospital of China Medical University, Shenyang, China; ^4^ Department of Anesthesiology, The Fourth Affiliated Hospital of China Medical University, Shenyang, China

**Keywords:** splenic metastasis, primary fallopian tube carcinoma, laparoscopic splenectomy, total hysterectomy, bilateral salpingo-oophorectomy

## Abstract

Metastases to the spleen from various non-hematologic malignancies are generally not a common clinical event and usually indicate the late dissemination of disease. Solitary splenic metastases from solid neoplasm are extremely uncommon. Furthermore, solitary metastasis to the spleen from primary fallopian tube carcinoma (PFTC) is extremely rare and has not been reported previously. We report a case of isolated splenic metastasis in a 60-year-old woman, occurring 13 months after a total hysterectomy, a bilateral salpingo-oophorectomy, a pelvic lymphadenectomy, a para-aortic lymphadenectomy, an omentectomy, and an appendectomy were performed for PFTC. The patient’s serum tumor marker CA125 was elevated to 49.25 U/ml (N < 35.0 U/ml). An abdominal computed tomography (CT) scan revealed a 4.0 × 3.0 cm low-density lesion in the spleen that was potentially malignant, with no lymphadenectasis or distant metastasis. The patient underwent a laparoscopic exploration, and one lesion was found in the spleen. Then, a laparoscopic splenectomy (LS) confirmed a splenic metastasis from PFTC. The histopathological diagnosis showed that the splenic lesion was a high-differentiated serous carcinoma from PFTC metastasis. The patient recovered for over 1 year, with no tumor recurrence. This is the first reported case of an isolated splenic metastasis from PFTC. This case underlines the importance of serum tumor marker assessment, medical imaging examination, and history of malignancy during follow-up, and LS seems to be the optimal approach for isolated splenic metastasis from PFTC.

## Introduction

Splenic metastasis is generally not a common clinical event and has an incidence of 0.6–1.1% in populations with carcinoma according to a large clinicopathologic study (1). Splenic metastasis of solid malignant tumors usually occurs in a range of extensive multiple-organ cancers and usually indicates the late dissemination of diseases. It is extremely rare to find that the spleen is the only site of metastatic spread (2). In a recent review of 29,364 patients with metastatic malignant tumors, Sauer et al. (3) found that the spleen is involved in approximately 1% of all metastases. The most common primary malignant tumors of splenic metastases are breast, lung, ovarian, and colorectal cancers, and melanomas and gastric carcinomas (1, 4–6). Notably, PFTC is the rarest tumor of the female genital tract and accounts for approximately 0.5% of all gynecologic carcinomas (7–9). Similarly, splenic metastasis from other tumors is infrequent, and solitary metastasis is also extremely rare (2, 10–13). Owing to its rarity and the lack of typical symptoms, splenic metastasis from PFTC presents a diagnostic dilemma. LS is the standard diagnostic procedure for many malignant and benign hematologic diseases (14, 15). Laparoscopic surgery reduces postoperative complications and has a faster recovery than an open operation (14–16). However, evidence for the application of LS in isolated splenic metastasis is limited (13, 17–20).

Here, we report an infrequent case of metastatic PFTC to the spleen. Furthermore, we review the clinical characteristics of an isolated splenic lesion and discuss diagnostic considerations and therapeutic strategies for improving the clinic management and survival time of patients.

## Case description

**Figure 1:**
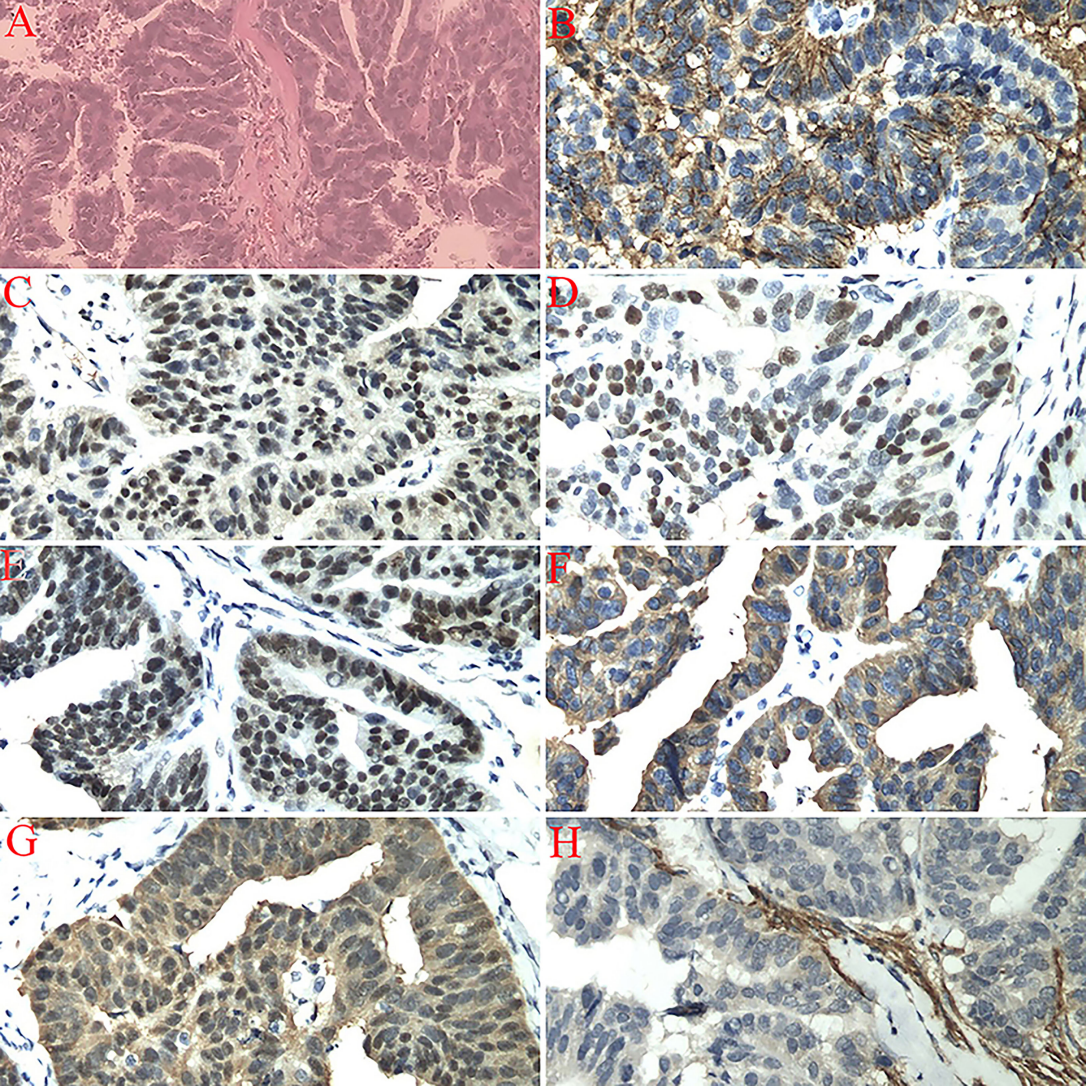
**(A)** Left fallopian tube invasive serous carcinoma (high-grade), bilateral ovaries, uterus, cervix, omentum, right fallopian tube, appendix, and dome margin with no tumor tissue (H&E staining, 200×). **(B)** Immunostaining for CA125 showing a positive reaction (200×). **(C)** Immunostaining for CR showing a positive reaction (200×). **(D)** Immunostaining for P53 showing a positive reaction (200×). **(E)** Immunostaining for WT1 showing a positive reaction (200×). **(F)** Immunostaining for CKL showing a positive reaction (200×). **(G)** Immunostaining for P16 showing a positive reaction (200×). **(H)** Immunostaining for CD10 showing a negative reaction (200×).

A 59-year-old postmenopausal woman, who went through menopause at the age of 47, was admitted to the hospital because of pain in her left lower abdomen for 3 months. CT and transvaginal sonography found a mass in the left adnexal area. Serum tumor markers were assayed. CA125 antigen level was 1,483 U/ml (N < 35.0U/ml); CEA, CA724, and AFP levels were normal. The results of a pap smear and colonoscopy were normal. The patient was counseled and underwent an exploratory laparotomy. The left adnexa showed an encapsulated solid mass in the ampullary portion of the left tube, with a size of 4.0 × 3.0 × 3.0 cm. Both ovaries and right adnexa were normal. The omentum and parietal and visceral peritoneum looked grossly normal. Total hysterectomy, bilateral salpingo-oophorectomy, pelvic lymphadenectomy, para-aortic lymphadenectomy, omentectomy, and appendectomy were performed. Histological examination of the resected specimen revealed a high-differentiated serous carcinoma of the left fallopian tube with serosa invasion (**Figures 1A–H**). The uterus, both ovaries, right fallopian tube, omentum, lymph nodes, and the washing fluid were free of tumor. The patient was diagnosed with PFTC Stage IC according to the International Federation of Gynecology and Obstetrics (FIGO) 2006 staging system. After the operation, the patient received six cycles of chemotherapy (150 mg of carboplatin and 100 mg of docetaxel). Two months after adjuvant chemotherapy, there was no evidence of metastatic lesions of the tumor and CA125 was normal through the follow-up.

**Figure 2:**
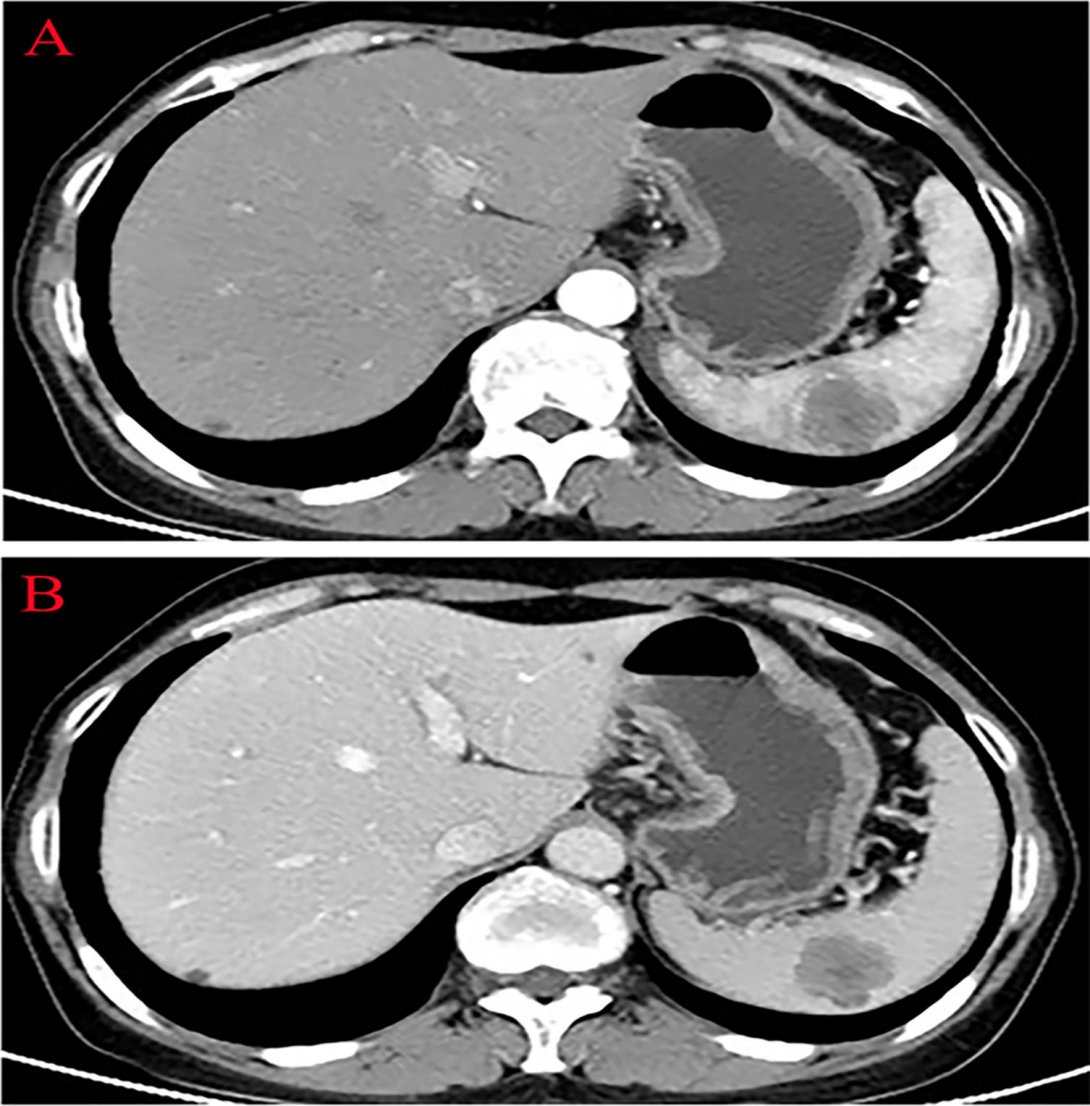
Abdominal computed tomography showing a low-density lesion in the low pole of the spleen with contrast enhancement during the arterial phase. **(A)** Arterial phase. **(B)** Venous phase.

Thirteen months after PFTC surgery, the patient’s serum tumor marker CA125 increased to 49.25 U/ml (N < 35.0 U/ml). The abdominal CT scan revealed a 4.0×3.0 cm low-density lesion in the spleen that was potentially malignant, with mild contrast enhancement during the arterial and no lymphadenectasis or distant metastasis (**Figures 2A, B**). The patient had no symptoms at all. The hemoglobin and platelet counts were normal. Owing to the history of PFTC, the high risk for splenic bleeding, and the risk for tumor development and spontaneous rupture in the case of biopsy, the patient underwent a LS.

**Figure 3:**
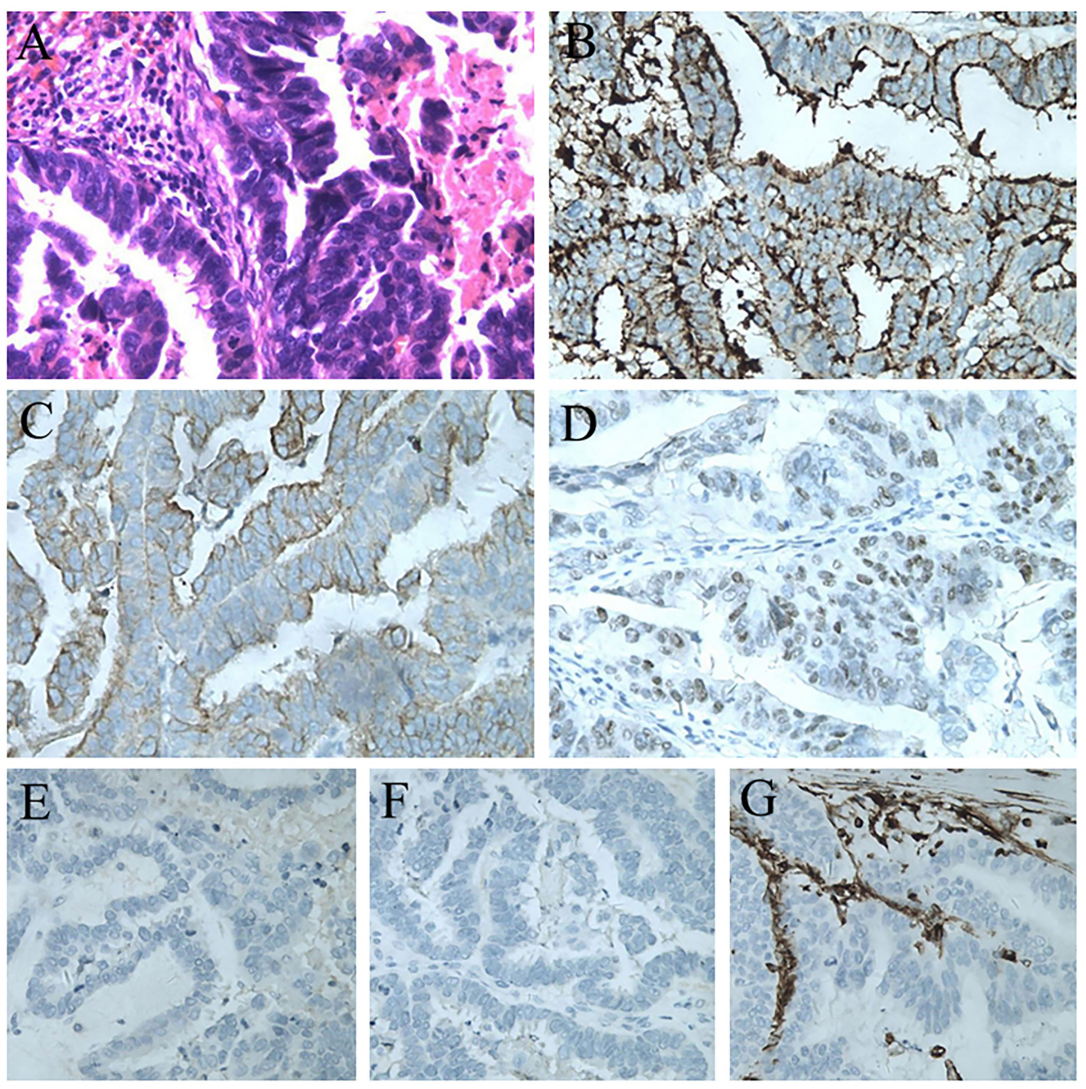
**(A)** Postoperative pathological result of the splenic lesion: serous carcinoma from the primary fallopian tube carcinoma (H&E staining, 200×). **(B)** Immunostaining for CA125 showing a positive reaction (200×). **(C)** Immunostaining for CK7 showing a positive reaction (200×). **(D)** Immunostaining for ER showing a positive reaction (200×). **(E)** Immunostaining for NapsinA showing a negative reaction (200×). **(F)** Immunostaining for Inhibin-α showing a negative reaction (200×). **(G)** Immunostaining for Vimentin showing a negative reaction (200×).

The splenic lesion was verified by surgical exploration; no other organs were involved and no peritoneal dissemination was found. The specimen showed one lesion, measuring 5.5 × 3.5cm, located at the lower pole of the spleen. The tumor was yellowish-white and clearly different from the adjacent splenic parenchyma. The histopathological diagnosis confirmed the splenic metastasis from PFTC (**Figure 3A**), supported by immunohistochemical stains positive for CA125, CK-7, and ER and negative for NapsinA, Inhibin-α, and Vimentin (**Figure 3B–G**).

There were no postoperative complications. The patient underwent six cycles of adjuvant chemotherapy after the operation and has been disease-free after the splenectomy with a good performance status up to 1 year after the LS.

## Discussion

PTFC is the rarest tumor of the female genital tract and accounts for approximately 0.5% of all female tract cancers (7–9). Recently it has been proposed to be the origin of the majority of pelvic or ovarian high-grade serous adenocarcinomas (21). PFTC spreads by means of local invasion, transluminal migration, and the bloodstream. Meanwhile, PFTC is richly permeated through the infundibular pelvic lymphatics and drains into the para-aortic lymph nodes (22). The CA125 antigen level is, nevertheless, indicative of poor prognosis, and can be used as a marker of disease recurrence. The pathological types of carcinoma are serous carcinoma and endometrioid carcinoma, followed by undifferentiated, clear cell, mucinous, and transitional carcinoma (22–24). In this case, the serum CA125 of the patient was elevated and the pathological type was serous carcinoma.

As we all know, the spleen is generally an organ that is hostile to malignant tumor implantation, which explains the low incidence rate of metastatic lesions in the spleen reported in the literature (2–5). Considering the primary lesion is located in the left fallopian tube, which is close to the spleen anatomically, the malignancy carcinoma of the fallopian tube in this case involved the spleen.

A long-term clinicopathological study summarized several reasons why metastatic diseases involving the spleen may not be easy to identify. First, many splenic metastases are asymptomatic, which makes diagnosis more difficult. Second, some splenic tumors in the study were too small to be readily identified during surgical exploration. Third, some tumors are not multiple, but solitary or diffuse, and therefore, would be confused with primary splenic tumors, such as hamartomas or lymphomas (1).

Splenic metastasis is most often incidentally detected by ultrasonography (US) or CT during follow-up of tumor patients (20). In a study comparing preoperative ultrasound and CT in the diagnosis of splenic metastasis after surgery, it was found that both methods had high specificity, sensitivity, and accuracy. However, US showed false positive results in the micronodule pattern of spleen involvement (25). Meanwhile, Sen et al. (26) reported that the use of techniques with high specificity and accuracy in the diagnosis of splenic metastasis in patients with malignant melanoma, such as positron emission tomography (PET) or CT, will improve the survival rate after an earlier splenectomy. However, CT, MR, US, radionuclide scans, and PET imaging cannot distinguish metastasis from other splenic diseases, lymphoma, or infection. Cytological examination is required as it may affect prognosis and treatment decisions (27). With the improvement and development of the guidance mode of percutaneous biopsy, a retrospective analysis showed that the overall accuracy rate in 160 patients who received splenic biopsy without complications was 98.1% (28). The indications of splenic biopsy are expanding, and the results showed that splenic fine-needle aspiration biopsy is a safe, cheap, and effective diagnostic technique. Therefore, it should be considered as a safer and first-line option in the evaluation of splenic lesions (18, 29). This case was completely asymptomatic and a splenic lesion was detected accidentally by abdominal CT in the follow-up. The patient refused fine-needle aspiration biopsy of the splenic lesion due to the complications. Taking the history of PFTC and raised serum CA125 level into account, this case was diagnosed with splenic metastasis from PFTC, which was eventually confirmed by pathological and immunohistochemical examination.

The treatment accepted worldwide is the classic splenectomy. Some studies have proposed that splenectomy is the proper therapeutic modality for an isolated splenic metastasis (30, 31) and long-term survival can be achieved by splenectomy for spleen-only metastasis (20). LS is the standard diagnostic procedure for many malignant and benign hematologic diseases (14, 15). The application of laparoscopy can decrease postoperative complications, including wound infections, pneumonia, and atelectasis, and enables patients to be discharged from hospital more quickly after surgery (14–16). However, the evidence for LS in isolated splenic metastases is limited (13, 17–19). The patient in this case underwent LS successfully without any complications, such as infection, intraperitoneal hemorrhage, or pancreatic injury.

## Conclusion

Isolated splenic metastasis from PFTC is an extremely rare finding during follow-up. This is the first case of isolated splenic metastasis from PFTC. This case will provide the basis for the prevention and diagnosis of this rare clinical disease and improve the treatment and survival time of patients. Close monitoring of patients with PFTC after primary treatment is conducive to the early diagnosis of such a metastasis. This case underlines the importance of serum tumor marker assessment, medical imaging examination, and history of malignancy during follow-up. LS seems to be the optimal approach for isolated splenic metastasis from PFTC.

## Data availability statement

The original contributions presented in the study are included in the article/supplementary material. Further inquiries can be directed to the corresponding authors.

## Ethics statement

The studies involving human participants were reviewed and approved by Ethics Committee of the Fourth Affiliated Hospital of China Medical University. The patients/participants provided their written informed consent to participate in this study. Written informed consent was obtained from the individual(s) for the publication of any potentially identifiable images or data included in this article.

## Author contributions

DL and XJ contributed to the conception and design of the study. DK and DZ wrote the first draft of the manuscript and prepared the figures. DL and XJ critically revised the manuscript for intellectual content. All authors contributed to manuscript revision and read and approved the submitted version.
